# Integration Analysis of m6A Related Genes in Skin Cutaneous Melanoma and the Biological Function Research of the SPRR1B

**DOI:** 10.3389/fonc.2021.729045

**Published:** 2021-10-19

**Authors:** Shupeng Shi, Zhihua Fan, Yang Liu, Chengyu Huang, Jianda Zhou

**Affiliations:** ^1^ Department of Plastic Surgery, The Third Xiangya Hospital, Central South University, Changsha, China; ^2^ XiangYa School of Medicine, Central South University, Changsha, China

**Keywords:** skin cutaneous melanoma, bioinformatics, m6A, immune infiltration, tumor metastasis, SPRR1B

## Abstract

Melanoma has gradually entered the public view because of its high morbidity and rising prevalence rate, which is a serious threat to human life and health. Recently, N6-methyladenine (m6A) modification has been increasingly confirmed as a potential role in the development of tumogenesis. The purpose of this study is to explore the role and function of m6a-related regulators in the development of melanoma disease at the molecular, cellular and clinical levels through bioinformatics and traditional experiments. We screened and validated differential expression genes (DEGs) in m6A regulators *via* the GEO, GTEx, TCGA database. The biological processes and signaling pathway involved by DEGs were improved by constructing bioinformational methods such as PPI, GO enrichment, KEGG enrichment, GSEA enrichment, and immune infiltration analysis. And then, we explored the biological function of the key gene, SPRR1B, through cell invasion, migration, infiltration, and tissue chips. The gene IGF2BP3 which was differentially expressed in m6A regulatory factor gene was screened. The results of the enrichment analysis are significantly enriched in the biological processes and pathways of the skin barrier, epidermal differentiation, cytoskeleton, lymphocyte migration and other pathways, pointing to the direction of tumor immunity and tumor metastasis. Tumor immune-related genes YTHDC1, YTHDC2 and ALKBH5 were found. Knock SPRR1B reduction group had a significantly lower invasive ability, the ability to migrate. Nomogram prediction model shows that SPRR1B increased, expressing a worse prognosis. For this purpose, the relationship between m6A regulatory factor and melanoma progression was explored. At the same time, it was found that the abnormal up-regulated expression of SPRR1B before metastasis would lead to poor prognosis of melanoma. SPRR1B promotes the proliferation, invasion and migration of human melanoma cells.

## Introduction

Melanoma is one of the deadliest and most difficult to treat human tumors in the world. Melanoma accounts for more than 75% of skin cancer deaths, and the 5-year relative survival rates of patients in local and regional melanoma were 98% and 64%, respectively (1). In recent years, the incidence of malignant melanoma has increased at an annual rate of 3% and 5%, making it the fastest growing malignant tumor in the world (2). Melanoma has the characteristics of concealed onset, rapid development, strong metastasis and poor prognosis, which is a serious threat to human health and life.

In recent years, many studies have proved that tumor epigenetic disorders, such as DNA methylation, non-coding RNA, histone modification, play an important role in the occurrence and development of tumors. N6-methyladenine (m6A), a dynamic and reversible epigenetic modification, regulates almost every step of RNA metabolism through m6A regulatory factors (methyltransferase, demethylase and methylated reading protein) (3, 4). With the rapid development of high-throughput sequencing technology and the gradual development of epigenetic research, the function of m6A modification in different biological processes has attracted more and more attention. It has been confirmed that m6A modification is involved in regulating gene expression and plays an important role in a variety of biological processes. Previous studies on the complex mechanisms of m6A modification in human diseases, including cancer, have been reported (5–7). Dynamic m6A level can participate in tumor development by regulating the expression of tumor-related genes in different types of tumors (8).

Recently, more and more studies have shown that the imbalance of m6A regulatory factors may be involved in the progression of cancer. For example, in lung cancer, methyltransferase METTL3 is up-regulated and promotes the growth and invasion of cancer cells (9). In addition, in melanoma, some studies have found that demethylase FTO can promote the growth of melanoma. Knockout of fat mass and obesity-associated protein (FTO) can increase the m6A methylation of important genes such as PD-1,CXC chemokine receptor 4 (CXCR4) and SRYBox transcription factor 10 (SOX10) in melanoma cells (10). These studies suggest that m6A plays a potentially important role in the development of melanoma. However, m6A-related genes including m6A regulatory factors have been identified, and the potential relationship between m6A-related genes and melanoma development is still unclear.

In this study, we used Gene Expression Omnibus (GEO), The Cancer Genome Atlas (TCGA) and Genotype-Tissue Expression(GTEx) databases to obtain RNA transcriptome or clinical data of cutaneous melanoma and normal tissues. The results pointed to the direction of tumor immunity and tumor metastasis. We found that the most significant difference between cancer and paracancerous m6A regulatory factor is IGF2BP3. The results of its functional enrichment analysis point to the direction of tumor immunity and tumor metastasis. Then we analyzed the relationship between IGF2BP3 and other m6A regulatory factors and tumor immunity and metastasis. At the same time, in the previous data analysis, we observed that a metastasis-related gene SPRR1B appeared many times and was significantly negatively correlated with IGF2BP3. SPRR1B is one of the proline-rich small protein (smallproline-richproteins, SPRR) gene family (11). As a member of the SPRR family, the study of SPRR1B involves a variety of diseases, including cancer, conjunctival disease, and skin diseases (12, 13). We are interested in its potential role in metastasis, so we further analyzed the effects of SPRR1B on survival and prognosis and on proliferation, invasion and migration. Our results provide a new view for the comprehensive action of m6A regulatory factors and other m6A related genes in skin melanoma, and it is found for the first time that SPRR1B can promote the proliferation, invasion and migration of melanoma cells.

## Materials and Methods

### Dataset Selection and Description

The tissue samples of human skin melanoma were screened in the gene expression database GEO (http://www.ncbi.nlm.nih.gov/geo/), and two data sets of GSE15605 and GSE100050 were obtained. The chip platform was GPL570 [(HG-U133_Plus_2) Affymetrix Human Genome U133 Plus 2.0 Array] (14). Among them, the data set GSE15605 included 74 samples including 16 normal samples, 46 primary melanoma samples and 12 metastatic samples. There were 12 samples in data set GSE100050, including 6 normal samples and 6 melanoma samples. 127samples of metastatic cutaneous melanoma were obtained from TCGA database, 297cases of cutaneous melanoma were not metastasized, and the data of normal healthy people were obtained from GTEx database as supplementary control.

### Data Preprocessing and Difference Analysis

The data set was processed by the hgu133plus2.db package of R language, and normalized correction was carried out. The batch effect was removed from the data by using the normalize between arrays function of R language. The box diagram before and after correction was drawn, and the effect of pretreatment was observed. Limma package was used for differential analysis, and the screening criteria were | log FC | > 1 and p < 0.05. The differentially expressed genes related to m6A in skin melanoma and normal tissues were screened. Use the pheatmap package to draw the heat map. The differential genes were verified by online tool GEPIA (http://gepia.cancer-pku.cn/). The website, developed by a team at Peking University, combines data from TCGA and GTEX and can be used to compare differential expression between tumor and normal tissues.

### Enrichment Analysis (GO, KEGG)

Cluster Profiler package (15) in R language and GO plot package were used for GO enrichment analysis, KEGG enrichment analysis (16) and GO enrichment analysis visualization. According to the enrich GO function in cluster Profiler package was analyzed. BH (Benjamin correction method) was selected to correct the P value, and the GO circle function was used to draw the circle diagram of GO enrichment analysis.

### PPI Network Construction

In order to determine the interactions and cellular signals between proteins and their functions, the STRING online database (https://string-db.org/) used to search the interacting genes was used to systematically analyze the differential genes screened and verified in skin melanoma, and the PPI network was constructed and visualized (17).

### GESA Gene Enrichment Analysis

GO and KEGG enrichment analysis required screening for differences first, in order to avoid ignoring non-standard screening but contribute to other genes, we used Gene Set Enrichment Analysis (GSEA) (18) to identify the associated with the disease phenotype expressed genes or proteins to determine the relationship between this intervention and the occurrence of melanoma. The program which ran GSEA is the Java version of GSEA-3.0. jar, enriched with the Hallmark gene collection and KEGG gene collection.

### CIBERSORT Immune Infiltration Assay and WGCNA

Using the screened and preprocessed GSE15605 skin melanoma data, 46 primary skin melanoma samples were extracted as immune infiltration data set. The study used CIBERSORT.R to infer the content of immune cells based on the gene expression feature set LM22 of 22 known immune cell subtypes. An expression matrix of the percentage of Cibersort immune cells in each sample was extracted based on the results of immunoinfiltration analysis of skin melanoma carcinoma *in situ* in the GSE15605 data set. Corrplot package of R language was used for pair-to-pair correlation analysis of immune cell content. Using R language WGCNA (Weighted Gene Co-Expression Network Analysis) (19) package weighted gene express network analysis. Spearman correlation was used to describe the correlation between genes and immune cells, and the correlation between the m6A regulatory factor genes and the core genes in the co-expression network analysis was examined, as well as the correlation between the m6A regulatory factor genes and PD-L1 molecules.

### Cell Proliferation, Invasion, and Migration

The expression of SPRR1B was detected by real-time fluorescence quantitative PCR (qRT-PCR) in four human melanoma cell lines A375, SK-MEL-2, A2058 and SK-MEL-28, which were purchased from the cell bank of the Chinese Academy of Sciences (Shanghai, China). SPRR1B was highly expressed in A375 and SK-MEL-2 cell lines. The cell proliferation, invasion and migration experiments were carried out using the cells with SPRR1B knockdown gene. In order to detect cell proliferation, CCK-8 cell proliferation assay (Japanese Dojindo) was used. 48 hours after transfection, the cell suspension was laid in a 96-well plate at a density of 5000 cells per 100 μL, and the proliferation of CCK-8 cells was measured at 0, 24h, 48h, 72h and 96 h, respectively. The wavelength absorbance at 450nm was measured by enzyme labeling instrument (Thermo Scientific Corp), and the OD value was measured, and the cell growth curve was drawn. Repeat the experiment at least 3 times, 3 holes each time. Cell migration and invasion were detected by Transwell assay in Transwell chambers with or without matrix glue (Becton Dickinson Company, USA). The malignantly transformed cells at the rate of 10000 cells/well were inoculated into the upper part of the Transwell chamber, and the complete medium was added outside the well and incubated in the cell incubator for 20-24h. Cells were fixed with 5% paraformaldehyde, stained with crystal violet (0.5%), and 3 random cells (200x) with high magnification field were counted. All measurements must be carried out in at least triplicate and repeated at least three times independently.

### Tissue Microarrays

Tissue microarrays were constructed by Shanghai Zhuoli Biotechnology Co, Ltd (Zhuoli Biotechnology Co, Shanghai, China). TMA included primary cutaneous melanoma(8 cases), metastatic melanoma (10 cases) and normal skin (18 cases), one of the metastatic melanoma of TMA without no tumor tissue(**Supplementary Table 2** and **Supplementary Figure 1**). The most typical tumor area were identified by the hematoxylin–eosin labeling of paraffin blocks of the sample tissue. The core of each donor block with a 1.0mm diameter was transferred to the receptor block microarray layer and arranged in the receptor paraffin block.

### Statistical Analysis

The above data analysis and mapping were operated under R language environment and Java environment, and ImageJ software was used to count cells. Limma package was used for correlation analysis and difference analysis. Hypergeometric distribution and Fisher’s exact test were used for functional enrichment analysis, and BH (Benjamin correction method) was used for P-value correction. In all statistical tests, P value less than 0.05 was considered to be statistically significant.

## Results

### Differential Analysis of the Regulatory Factors of m6A in Cutaneous Melanoma

**Figure 1:**
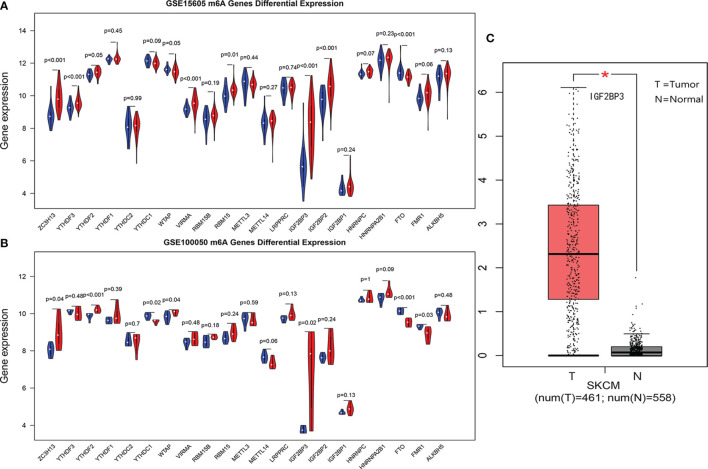
**(A, B)** are violin diagrams of GSE15605 and GSE100050 (red is the cancer group, blue is the normal group). **(C)** Expression of IGF2BP3 in skin melanoma and normal tissue in TCGA and GTEX data, where *P < 0.05.

In GEO data sets GSE15605 and GSE100050, the most significant common difference of m6A regulatory factors between tumors and normal tissues was IGF2BP3 (**Figures 1A, B**). The joint verification of TCGA and GTEx (**Figure 1C**) also showed that IGF2BP3 was significantly up-regulated in cutaneous melanoma.

### Functional Enrichment Analysis of IGF2BP3

**Figure 2:**
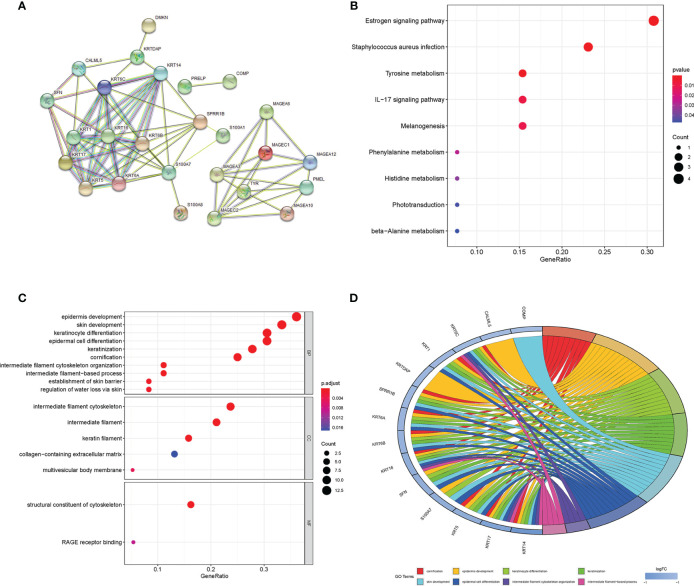
**(A)** Differential gene protein interaction networks; **(B)** KEGG enrichment bubble map of differential genes; **(C)** GO enrichment analysis bubble diagram of differential genes (color represents P value, the redder the color, the more significant the difference; The length of the histogram and the bubble size of the bubble graph represent the degree of enrichment) **(D)** GO enrichment circle diagram of the differential genes.

In the IGF2BP3 expression group of differential gene protein interaction network (**Figure 2A**), the left network was all down-regulated genes in the IGF2BP3 high expression group, mainly composed of KRT family members and SPRR1B, S100A7 and other genes. The right network was dominated by members of the MAGE family. Except for Tyr and PMEL, all of the high-expression groups expressed upregulated genes in melanoma.

The results of KEGG enrichment analysis (**Figure 2B**) and GO enrichment analysis (**Figures 2C, D**) showed that differential genes were significantly enriched in Estrogen signaling pathway, cornification, epidermal cell differentiation, intermediate filament cytoskeleton organization, establishment of skin barrier, and other signal pathways or biological processes.

In addition, we found that in the GO enrichment analysis of biological process (BP) classification, the differential genes were also significantly enriched in the biological processes related to immunity, such as regulation of lymphocyte migration, positive regulation of lymphocyte chemotaxis and lymphocyte migration.

**Figure 3:**
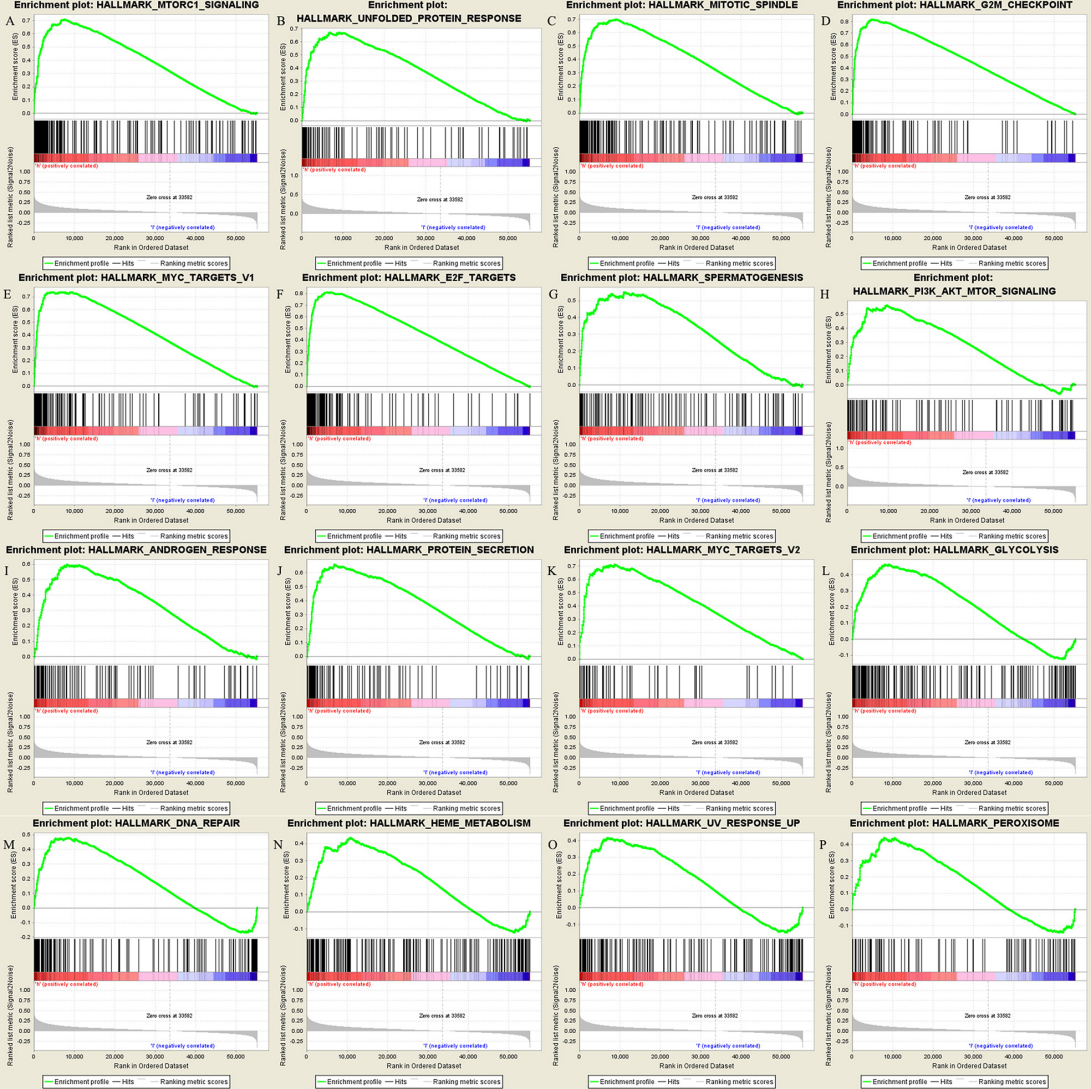
Results of GSEA enrichment pathways: **(A–P)** in order of significance, they were the top 16 significantly enriched pathways.

GSEA enrichment analysis (**Figure 3**) shows that the most significant pathway is MTORC1 signaling, which is closely related to tumor immunosuppression, tumor growth and metabolism (20). In addition, there are unfolded protein response of PI3K-AKT-MTOR signaling, mitotic spindle, G2M checkpoint, MYC targets V1, E2F targets, etc. the gene set genes of these pathways are significantly enriched under the high expression of IGF2BP3. The gene sets of these pathways were significantly enriched under the high expression of IGF2BP3.

The result of IGF2BP3 function enrichment analysis indicated that IGF2BP3 may be related to tumor immunosuppression and tumor metastasis, so the relationship between IGF2BP3 and other m6A regulatory factors in melanoma and tumor immunity and metastasis will be further analyzed in the follow-up.

### Association of m6A Regulatory Factor With Tumor Immune Cell Subtypes and PDL1

**Figure 4:**
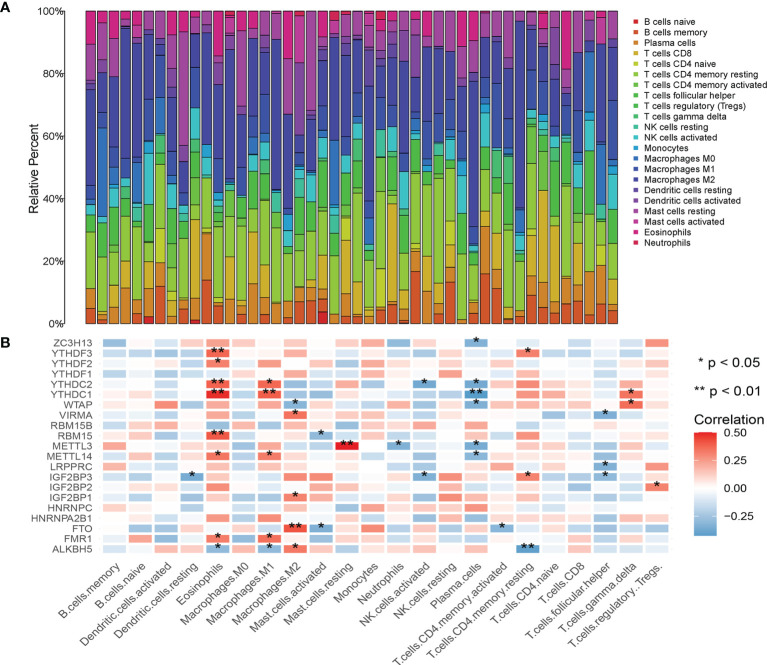
**(A)** Histogram of percentage of immune cell subtypes in skin melanoma samples; **(B)** Correlation analysis between m6A related genes and immune invasion.

**Figure 5:**
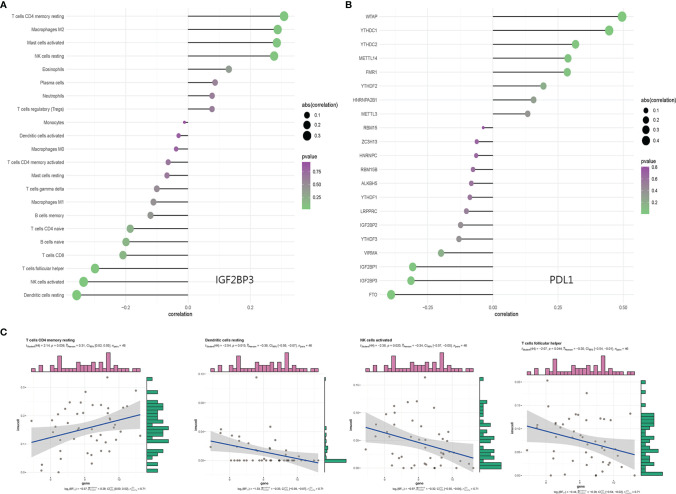
**(A)** Bar graph of IGF2BP3 and immunocyte subtype content; **(B)** Correlation analysis between m6A related genes and PDL1; **(C)** Correlation results of IGF2BP3 with resting dendritic cells, activated NK cells, follicular helper T cells, and resting CD4+ memory T cell subtypes.

The composition of 22 immune cell subtypes in each sample (**Figure 4A**) showed that in cutaneous melanoma carcinoma *in situ*, the top six immune cells with the highest infiltration level were M2 macrophages (21.55%), T cells CD4 memory resting (15.62%), resting mast cells (8.14%), follicular helper t cells (7.40%), M1 macrophages (7.04%), CD8+T cells (6.80%). The analysis of correlation between m6A regulatory factors and immune cell subtypes (**Figure 4B**) shows that IGF2BP3, YTHDC1, YTHDC2 and ALKBH5 are related to more immune cell subtypes. IGF2BP3 was significantly correlated with four immune cell subtypes (**Figures 5A–C**), which were positively correlated with resting CD4+ memory t cells and negatively correlated with resting dendritic cells, activated NK cells and follicular helper t cells. The analysis of the correlation between m6A regulatory factors and PD-L1 showed that PD-L1 was positively correlated with WTAP and YTHDC1-2, but opposite to FTO, IGF2BP3 and IGF2BP1.

### Constructing WGCNA Network

**Figure 6:**
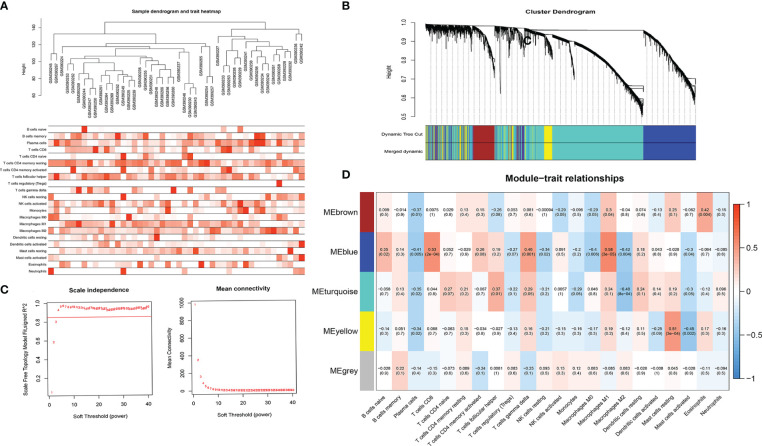
**(A)** Sample tree map and characteristic heat map; **(B)** Clustering diagram of gene modules; **(C)** Soft threshold screening graph, which respectively represents the correlation between soft threshold and R2 and the relationship between soft threshold and average connectivity (the higher the correlation coefficient squared, namely R2, the closer the network is to the scale-free network distribution, generally not less than 0.8; **(D)** Correlation results between co-expressed gene modules and immune cell subtype content.

**Figure 7:**
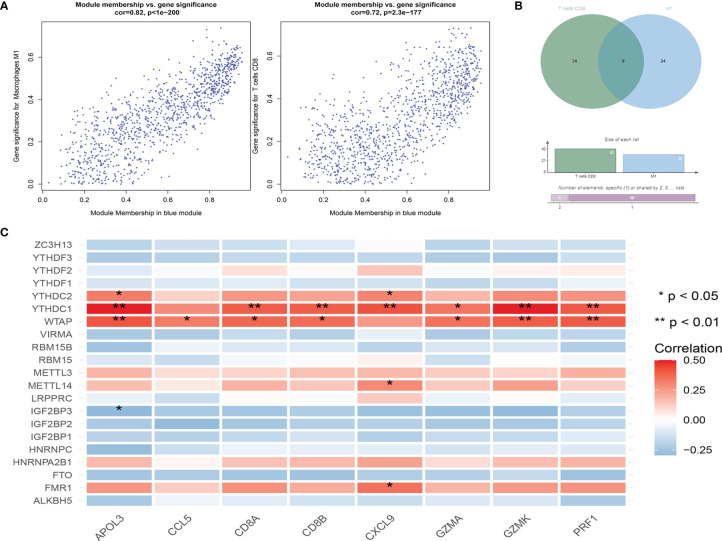
**(A)** Correlation results between gene expression and module characteristics of significantly related blue M1 macrophage module and blue CD8+T cell module; **(B)** Wien diagrams of significantly related module genes; **(C)** Correlation analysis between m6A related genes and common genes of significantly related modules.

We combined Cibersort immunoinfiltration data to construct a WGCNA network (**Figure 6**). We selected the most relevant module of network species cluster, the blue M1 macrophage module and the blue CD8+T cell module, for gene intersection and further correlation analysis with the m6A regulatory factor (**Figure 7**). Finally, 8 genes related to anti-tumor immunity were obtained, which were APOL3, CCL5, CD8A, CD8B, CXCL9, GZMA, GZMK and PRF1.We found that the m6A regulatory factors YTHDC1, YTHDC2, and WTAP were positively correlated with m6A, while IGF2BP3 was negatively correlated.

### Associations Between M6a Regulatory Factors and Metastatic Differential Genes.

**Figure 8:**
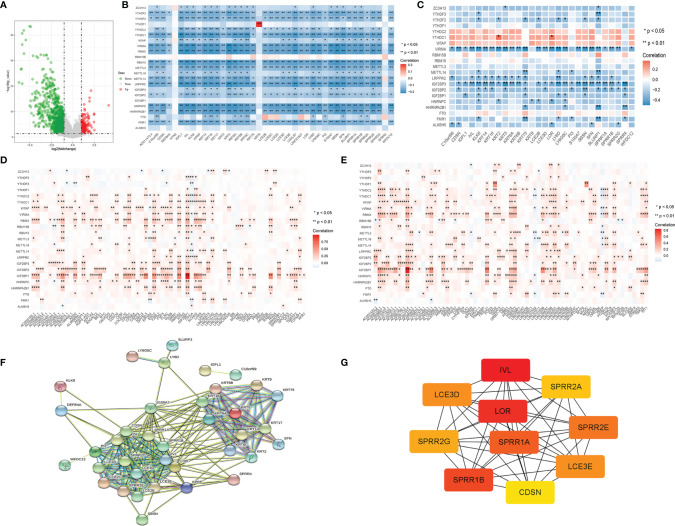
**(A)** Volcano map of metastasis-related differential gene screening; **(B)** log FC> 4 differences in gene regulation and m6A factor correlation analysis results; **(C)** log FC> 4 differences in gene regulation and m6A factor correlation analysis (GSE15605 dataset); **(D, E)** High differences in gene expression and metastatic carcinoma m6A regulation factor correlation analysis results; **(F)** log FC> 4 gene transfer difference of protein interaction network; **(G)** log FC> 4 transfer difference gene protein interaction networks at the core of the gene (the node color said correlation degree, the red associated with surrounding the high).

Differential gene screening was performed using TCGA skin melanoma carcinoma *in situ* and metastatic cancer data, and 135 genes were significantly up-regulated and 777 genes were down-regulated in metastatic cancer (**Figure 8A**) (**Supplementary Table 1**). The m6A regulatory factor was observed to be widely associated with the most significantly different gene (also significantly down-regulated) in metastatic cancers, with an overall negative correlation (**Figure 8B**) and opposite to the up-regulated gene (**Figures 8D, E**). In GSE15605 carcinoma *in situ* data, the overall trend was still negative correlated.; In addition, IGF2BP3 had the most significant correlation with VIRMA (**Figure 8C**). The most significantly different genes were used to construct a protein interaction network (**Figure 8F**). The network genes were mainly members of KRT family and SPRR family, and the most core genes in the network (**Figure 8G**) were IVL, LOR and SPRR1B.In addition, we found that the significantly down-regulated gene network in metastatic cancer was similar to the significantly down-regulated gene network in the IGF2BP3 overexpression group (**Figure 2A**), both of which included KRT family members and SPRR1B, etc.

We observed multiple occurrences of SPRR1B, one of the most significantly down-regulated genes in the IGF2BP3 overexpression group, and one of the most significantly down-regulated genes in metastatic cancer.

### Expression Verification and GSEA Analysis of SPRR1B

**Figure 9:**
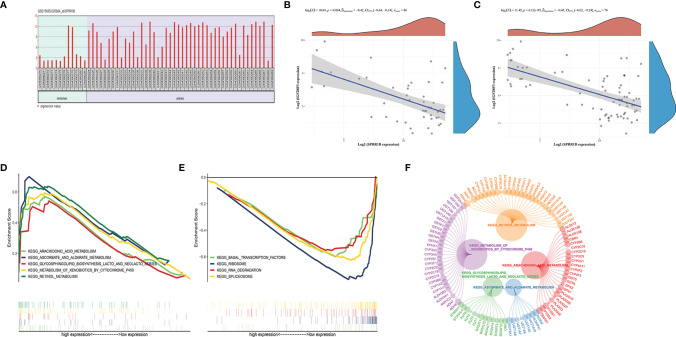
**(A)** Expression of SPRR1B in carcinoma *in situ* and metastatic carcinoma in the GSE15605 dataset; **(B, C)** Correlation between expression of SPRR1B and IGF2BP3 in carcinoma *in situ* and metastatic carcinoma; **(D, E)** SPRR1b GSEA enrichment score; **(F)** Upregulating the core genotype of each pathway (the circle of the gene indicates the contribution to the pathway, and the larger the circle indicates the greater the contribution of the gene).

We verified by GSE15605 data set that SPRR1B also showed high expression in melanoma carcinoma *in situ* (**Figure 9A**). In addition, in GSE15605 cancer *in situ* (**Figure 9B**), the correlation number between SPRR1B and IGF2BP3 expression was -0.42, p value was 0.004; as well as all cases including normal tissues and metastatic cancers (**Figure 9C**), the correlation coefficient between them is -0.45, and the p value is 6.12e-5, which is also consistent with the down-regulation of SPRR1B in IGF2BP3 high expression group.

GSEA enrichment analysis was performed for SPRR1B gene. The results showed that the five pathways were significantly enriched in the high superficial group of SPRR1B (**Figures 9D–F**). The four pathways were significantly enriched in the low expression group of SPRR1B (**Figure 9E**). The five up-regulated pathways are ascorbate and alginate metabolism, followed by Arachidonic acid metabolism. Among them, the most significant down-regulated four pathways are spliceosome.

### Effects of SPRR1B on Survival Prognosis and Proliferation, Invasion, and Migration

**Figure 10:**
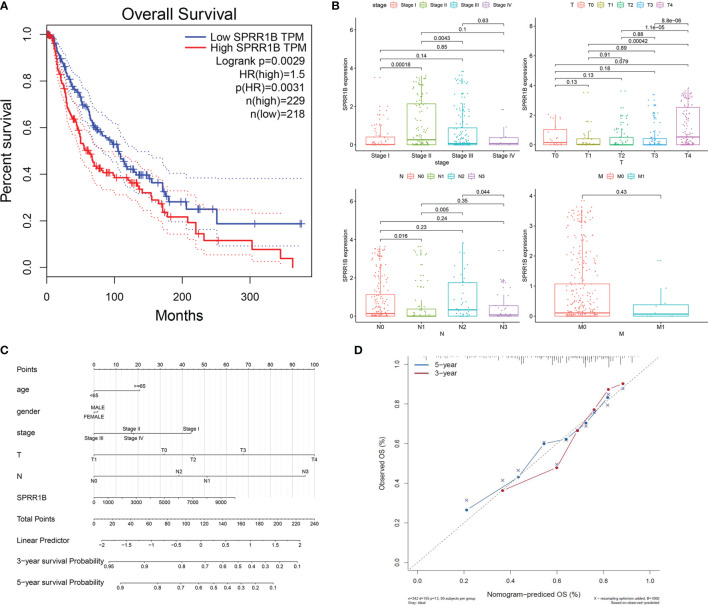
**(A)** Survival analysis results of SPRR1b; **(B)** Clinical correlation analysis of SPRR1B; **(C)** Clinical prediction model of SPRR1B by histogram; **(D)** Verification of calibration curve of line chart prediction model.

Survival analysis was performed on TCGA cutaneous melanoma clinical data set according to the expression level of SPRR1B (**Figure 10A**). It was observed that the overall survival of the group with high SPRR1B expression was significantly lower than that of the group with low SPRR1B expression (P = 0.0029). Nomogram prediction model (**Figure 10C**) showed that the survival risk increased with the increase of SPRR1B expression. The calibration curves (**Figure 10D**) showed that the actual 3 - and 5-year survival rates were close to the predicted values. We further performed a clinical correlation analysis (**Figure 10B**) based on TCGA data for cutaneous melanoma with TNM staging and clinical staging. We found that in the clinical stage, the two stages with the most obvious high expression enrichment were stage II and stage III. In the classification of T (primary foci), the two stages with the most obvious high expression enrichment were T0 and T4.In the classification of N (lymph node metastasis), the two stages with the most obvious high expression and enrichment were N0 and N2.In the classification of M (distant metastasis), the most significant high expression enrichment of M0 and M1 was M0.We observed an interesting phenomenon that SPRR1B was not expressed or low expressed in most cases, and the high expression and significant enrichment of SPRR1B always precede the development of more aggressive metastasis, so SPRR1B was significantly down-regulated in metastatic cancer.

**Figure 11:**
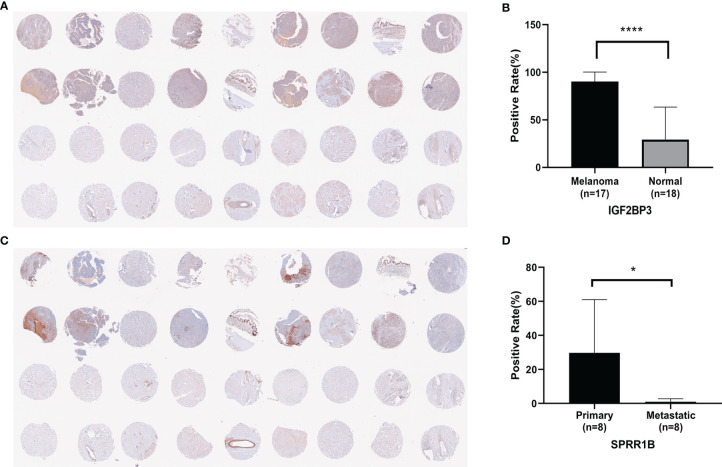
**(A)** Immunoreactivity of IGF2BP3 in human melanoma and normal skin tissue; **(B)** Positive rate of IGF2BP3 in human melanoma and normal skin tissue, where *P < 0.05; **(C)** Immunoreactivity of SPRR1B in human melanoma and normal skin.; **(D)** Positive rate of SPRR1B in primary melanoma and metastatic melanoma, where ****P < 0.0001.

After exclusion (**Supplementary Table 2**), 17 cases of melanoma were positive for IGF2BP3 and had a high positive rate, and 8 cases in normal skin tissues (50%) were weakly positive for IGF2BP3 (**Figure 11A**).The difference between the positive rate of IGF2BP3 in melanoma and normal skin was seen(**Figure 11B**).7 cases of primary melanoma (87.5%) were positive for SPRR1B, and 2 cases in metastatic melanoma (25%) were very low weakly positive for SPRR1B (**Figure 11C**).The difference between the positive rate of SPRR1B in Primary melanoma and metastatic melanoma was seen in **Figure 11D**.

**Figure 12:**
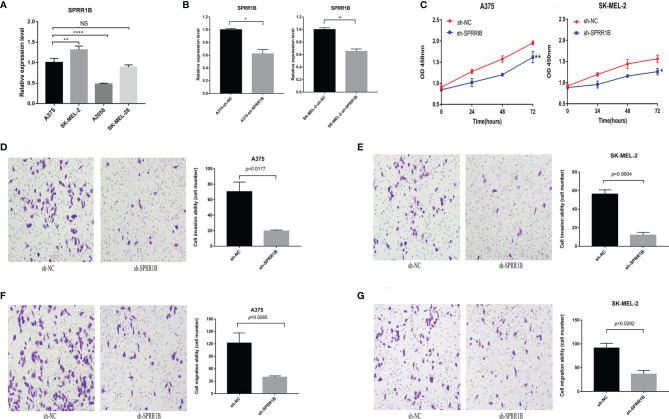
**(A)** the relative expression of SPRR1B gene in four melanoma cell lines, where *P < 0.05; **P < 0.01; ****P < 0.0001; NS, not statistically significant; **(B)** the expression of SPRR1B gene in knock-down group and contrast group in a375 cell line and SK-MEL-2 cell line; **(C)** CCK8 proliferation experiment results of C)A375 cell line and SK-MEL-2 cell line; **(D)** A375 cell knockdown SPRR1B genome and sh-NC group cell invasion experiment results (200 times field of vision) and statistical results; **(E)** SK-MEL-2 cell knockdown SPRR1B genome and sh-NC group cell invasion experiment results and statistical results; **(F)** The experimental results and statistical results of A375 cell knockdown SPRR1B genome and sh-NC group cell migration; **(G)** experimental results of skmel-2 cells knocking down SPRR1B genome and sh-NC group cell migration.

The expression of SPRR1B in A375, SK-MEL-2, A2058 and SK-MEL-28 human melanoma cell lines was detected by real-time fluorescence quantitative PCR (**Figure 12A**). It was found that the expression of SPRR1B in A375 and SK-MEL-2 was relatively high. The expression of the SPRR1B gene in sh-SPRR1B and sh-NC was then detected by qRT-PCR (**Figure 12B**). The results showed a significant reduction in SPRR1B gene expression in A375 (P<0.05) and SK-MEL-2 (P<0.01) cells compared to the control group (P <0.05). After SPRR1B gene knockout, we used CCK-8 proliferation assay to detect the number of human melanoma cells A375 and SK-MEL-2 and draw the growth curve (**Figure 12C**). It was observed that the proliferation of A375 and SK-MEL-2 knockout group (sh-SPRR1B) was significantly lower than that of the control group (sh-NC). SPRR1B knockout inhibited the proliferation of human melanoma cells.

Using Transwell microventricular experiments to detect the effect of SPRR1B genes on the invasion ability of A375 and SK-MEL-2 cells (**Figures 12D, E**), we found that the invasion ability of both A375 and SK-MEL-2 cells was significantly reduced compared to sh-NC cells. For A375 cells, the average number of sh-NC group was 70.3 ± 10.1 per 200x mirror field, while the average number of sh-SPRR1B group was 19.7 ± 0.9 per 200x visual field(P=0.0177). The average number of SK-MEL-2 cells in sh-NC group was 56.3 ± 3.7 per 200x visual field, while the average number of cells knocking out SPRR1B was 12.0 ± 2.5 per 200x visual field(P=0.0004). We used Transwell chamber test to detect the effect of SPRR1B gene on the migration ability of two human melanoma cell lines (**Figures 12F–G**). It was found that the migration ability of A375 and SK-MEL-2 cells in sh-SPRR1B group was significantly lower than that in sh-NC cells. For A375 cells, the average number of cells in sh-NC group was 122.0 ± 19.9, while the average number of sh-SPRR1B group was 39.7 ± 2.5(P=0.0256) per 200x lens visual field. The average number of SK-MEL-2 cells in sh-NC group was 91.3 ± 8.2 per 200x visual field, while the average number of sh-SPRR1B group was 36.7 ± 5.9 per 200x visual field, P=0.0282.

## Discussion

Globally, the incidence of melanoma is on the rise, with approximately 91,270 new cases of melanoma and 9,320 deaths due to melanoma each year, according to the latest melanoma report from the US National Cancer Institute (21). From traditional surgery and chemotherapy to immunotherapy in recent years, the treatment of melanin is also gradually developing. Immune checkpoint inhibitor (ICI) therapy has ignited a new hope of “cure” for melanoma, especially for advanced melanoma. however, various kinds of resistance caused by treatment, and a series of toxic reactions induced by inflammation are also increasing due to systemic administration. More and more immune-mediated adverse reactions have been reported (22), the development of targeted therapeutic strategies based on specific therapies is urgent. Many studies have shown that m6A modification is related to tumor proliferation (23), tumorigenesis, differentiation, invasion (9) and metastasis (24). Therefore, integrated analysis of m6A related genes in melanoma and exploring the function of genes in melanoma cells may become a new therapeutic target.

Through the screening of two sets of GEO data sets, we found that IGF2BP3 was significantly up-regulated in cutaneous melanoma. Differentially expressed genes of IGF2BP3 were screened, up-regulation mainly involves members of MAGE family, while down-regulation involves members of KRT family and other genes such as SPRR1B. As biomarkers in cancer and immunotherapy, MAGE gene family has attracted more and more attention and is abnormally expressed in possibly immunogenic cancers (25). MAGE family is associated with poor prognosis of tumor patients. For example, in non-small cell lung cancer, the expression of MAGEA9 is significantly correlated with decreased survival rate (26). Existing studies have also shown that MAGEs is the driving factor of tumorigenesis and development. *In situ* xenografts of human thyroid cancer cells overexpressing MAGEA3 increased the ability of tumor growth and metastasis to the lung (27), while MAGEC knockdown delayed the formation of metastatic melanoma *in vivo* (28). IGF2BP3, KRT family members and SPRR1B genes are the core genes enriched by GO. These results suggest that IGF2BP3 may participate in the process of tumorigenesis and development by stabilizing its mRNA. Another differential gene module, the main body of the KRT family, is the intermediate protein for epithelial cells to form intermediate filaments, which is widely used as a diagnostic biomarker in cancer (29, 30). The KRT family is closely related to the occurrence and metastasis of a variety of tumor types, including lung (31), breast cancer (32) and colon cancer (33). Moreover, KRT8 has also been reported to be abnormally expressed in melanoma (34). All the above evidence suggests that IGF2BP3 may be involved in the occurrence and development of melanoma by regulating the mRNA stability of these gene families and other genes.

The family of insulin-like growth factor 2mRNA binding proteins (IGF2BPs, including IGF2BP1/2/3) can identify m6A, block the degradation of target mRNA and promote mRNA translation in an m6A-dependent manner (35). We found that the results of GO, KEGG and GSEA function enrichment were significantly enriched in biological processes and pathways such as cell junction assembly, skin barrier, cytoskeleton, epidermal differentiation, lymphocyte migration, regulation of lymphocyte chemotaxis, mTORC1 signaling pathway and PI3K-Akt signaling pathway, and ultimately pointed to the direction of tumor immunity and tumor metastasis. Confirmed by CIBERSORT immune infiltration analysis, IGF2BP3 is indeed one of the m6A regulatory factors with the largest number of immune cell subtypes. In addition, we found that IGF2BP3 is one of the two m6A regulatory factors with the most significant correlation with metastasis-related genes in the GEO carcinoma *in situ* data set.

Studies have found that IGF2BP3 is closely associated with the occurrence of leukemia (36), atypical hyperplasia of Barrett’s esophagus (37), pancreatic duct neoplasia (38) and atypical endometriosis (39), etc. These evidences indicate that IGF2BP3 may play an important role in the occurrence of tumors. In addition, compared with normal tissues, IGF2BP3 was up-regulated in many tumors, including lung adenocarcinoma, ovarian cancer, breast invasive cancer, bladder urothelial carcinoma and so on (40). These results are consistent with the results of our study, suggesting that IGF2BP3 may play an important role in the occurrence and development of cutaneous melanoma.

GSEA enriched the gene expression after IGF2BP3 grouping, among which MTORC1 signal pathway and PI3K-AKT-MTOR signal were the most significant ones. In the past decades, we know that the characteristics of cancer include, etc., and the activation of mTOR signal is related to every cell carcinogenesis process, such as uncontrolled cell proliferation, escaping anti-tumor immunity and abnormal angiogenesis. The mTORC1 signal is involved in the growth and metabolism of tumor. there is evidence that mTORC1 regulates aerobic glycolysis by increasing the translation of hypoxia inducible factor (HIF)-1α. The activity of mTORC1 is also related to immunity, among which it is highly related to Th1 differentiation and anti-tumor immunity. PI3K signal can indirectly activate mTORC1 through AKT (41). Recent studies have shown that the activity of IGF2BP3 may be affected by mTOR, which is one of the main downstream effectors of phosphoinositide 3 kinase (PI3K) (42). Therefore, it is observed that the up-regulation of IGF2BP3 expression is consistent with the up-regulation of mTORC1 signal including PI3K-AKT-MTOR signal pathway, which plays a synergistic role in the growth and metabolism of skin melanoma and tumor immunity. These results indicate that IGF-2 BP-3 is involved in biological processes such as tumor proliferation and invasion and tumor immune infiltration in skin melanoma.

Tumor immunity is a new hot spot in the treatment of melanoma. m6A modification not only has great potential in tumor therapy, but also is related to tumor immunotherapy resistance. Our results showed that M2 macrophages, resting CD4+ memory T cells, resting mast cells, follicular helper T cells, M1 macrophages and CD8+T cells were abundant in cutaneous melanoma *in situ*. It has been reported that the levels of M1 macrophages and Th1 lymphocytes are positively correlated with prognosis and survival time (43–46). We found that the m6A regulatory factor related to anti-tumor immunity was significantly positively correlated with the gene PD-L1 involved in tumor immune escape, which is contradictory according to common sense. However, this finding supports many studies that the high expression of PD-L1 in cutaneous melanoma has a better prognosis. We believe that this precisely illustrates the adaptive immune resistance of PD-L1 expression in skin melanoma, because PD-L1 expression is associated with anti-tumor CD8+T cell response. In addition, as there are some contradictions in the current research on PD-L1 in melanoma, future studies should consider the influence of PD-L1 expression in time and space, as well as the interaction with anti-tumor immunity.

Our study showed that in tumor immunity, the most m6A regulatory factors associated with immune cell subtypes were YTHDC1, YTHDC2, WTAP and FTO, etc. In the results of WGCNA co-expression network, YTHDC1, YTHDC2 and WTAP were positively correlated with the screened anti-tumor immune genes, and it was widely statistically significant. In addition, WTAP, YTHDC1 and YTHDC2 are widely positively correlated with anti-tumor immune-related genes, while IGF2BP3 is widely negatively correlated with these genes, which may be involved in the immune escape of skin melanoma cells.

Metastatic cutaneous melanoma is associated with decreased survival and high mortality. Previous studies have looked at the difference between primary and metastatic melanoma (47). The mechanism of metastasis involves multiple pathways, including epithelial-mesenchymal transformation, angiogenesis, and invasion (48). Among the first 20 down-regulated genes in metastatic cancer, the down-regulated genes in PPI network are mainly KRT family members, SPRR1B genes, and so on. This result is very similar to the PPI network in which the most significant difference-related genes (| log FC | > 4) are transferred, and these are two different data sources.

Existing studies have shown that the SPRR family is associated with epidermis and tumors. Previous studies have found that the SPRR family can act as SH3 domain ligands to increase resistance to injury and is related to (EMT) in epithelial-mesenchymal transformation in bile duct cells (49). Some scholars (50) believe that the upregulation of SPRR is often accompanied by damage to the normal structure of the stratum corneum, which affects the function of epithelial barrier and leads to the decrease of skin hydration, antioxidation, wound healing, drug sensitivity and anti-infection ability. At the same time, some studies have shown that the expression of SPRR1B is down-regulated in metastatic melanoma (51). The results of TMA also confirmed that IGF2BP3 was significantly up-regulated in melanoma, and also confirmed that SPRR1B was significantly down-regulated in metastatic cancer before tumor invasion and metastasis.

In addition, some studies have shown that the loss of SPRR1B expression is accompanied by irreversible malignant changes (52). We preliminarily believe that it may play a potential role in metastasis, so we further analyze the effects of SPRR1B on survival and prognosis and on proliferation, invasion and migration. We found that SPRR1B is generally not expressed, but only up-regulated before metastasis, which can improve the ability of cell proliferation, invasion and migration to a certain extent. Previous studies (53) have shown that SPRR1B gene is overexpressed in oral squamous cell carcinoma stem cell-like cells, which affects cell growth. SPRR1B may be related to tumor cell growth by regulating MAP kinase signal transduction pathway. For skin melanoma, the mechanism of SPRR1B overexpression regulating tumor invasion and metastasis may also go through the pathway mentioned above, but more experimental data are needed to verify this conjecture.

SPRR1B gene enrichment analysis showed that the high expression group enriched the first ascorbic acid and alginate pathway, and it was found that the metabolites of ascorbic acid and alginate were different before and after skin melanoma metastasis, which may be related to the metastasis progress (54). The high expression of SPRR1B has a poor prognosis and plays an important role in the prognostic model, which shows its potential as a good prognostic marker for cutaneous melanoma. However, more experiments are needed to verify whether SPRR1B and its related genes can be used as therapeutic targets.

We demonstrated for the first time that SPRR1B can promote the proliferation, invasion and migration of melanoma cells, providing a new perspective on the combined role of m6A regulatory factors and other m6A-related genes in cutaneous melanoma. Previous studies have revealed non-coding drivers of spliceosomal RNA, which are involved in abnormal splicing mechanisms in cancer, and may be a new therapeutic target (55). In the GSEA enrichment of this study, we also found the splice pathway with significant enrichment of low expression, and whether there is a certain relationship between it and the down-regulation or non-expression of SPRR1B needs to be further explored.

## Conclusion

In this study, we screened out the genes with common and significantly differentially expressed m6A regulatory factor genes, and found that IGF2BP3 was significantly higher expressed in skin melanoma than in normal tissues, and IGF2BP3 plays an important regulatory role in the occurrence and development of skin melanoma. In tumor immunity, YTHDC1, YTHDC2 and ALKBH5 were the most correlated regulatory factors of m6A with immune cell subtypes, and YTHDC1 and YTHDC2 were positively correlated with the selected anti-tumor immune genes. In the case of tumor metastasis, the m6A regulatory factor genes were negatively correlated with the significantly down-regulated genes in the metastatic cancer, while the up-regulated genes were positively correlated. SPRR1B was also found to be associated with the prognosis of melanoma. High expression of SPRR1B was associated with poor prognosis and was usually abnormally up-regulated before metastasis. SPRR1B promoted the proliferation, invasion, and migration of human melanoma cells.

## Data Availability Statement

The original contributions presented in the study are included in the article/**Supplementary Material**. Further inquiries can be directed to the corresponding author.

## Author Contributions

SS was a major contributor to all the experimental work, data analysis, and manuscript writing. ZF was involved in the experimental work. YL and CH were involved in data analysis. JZ conceptualized the project, acquired funding, and assisted with manuscript development. All authors read and approved the final manuscript.

## Funding

The current study was funded by the National Natural Science Foundation of China (No.81872219).

## Conflict of Interest

The authors declare that the research was conducted in the absence of any commercial or financial relationships that could be construed as a potential conflict of interest.

## Publisher’s Note

All claims expressed in this article are solely those of the authors and do not necessarily represent those of their affiliated organizations, or those of the publisher, the editors and the reviewers. Any product that may be evaluated in this article, or claim that may be made by its manufacturer, is not guaranteed or endorsed by the publisher.
