# Supplementary figures and images for: Integration Analysis of m6A Related Genes in Skin Cutaneous Melanoma and the Biological Function Research of the SPRR1B
Source: Front Oncol. 2021 Oct 19;11:729045. doi: 10.3389/fonc.2021.729045 (PMC8560968; doi:10.3389/fonc.2021.729045)

**Supplementary Figure 1.** The location of TMA


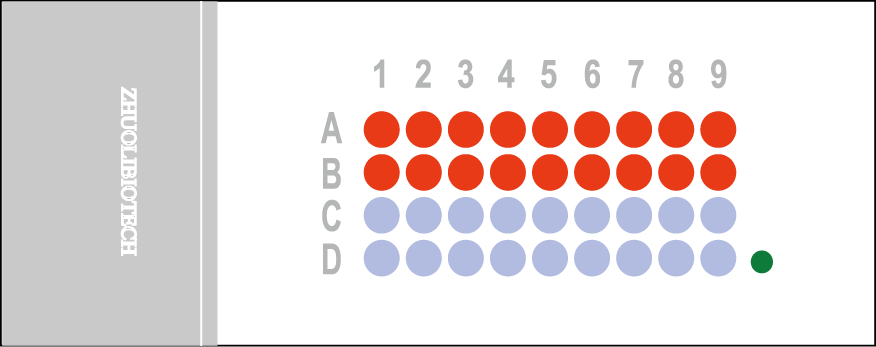

Supplement: Supplementary file 1 [file DataSheet_1.docx]
