# Supplementary material for: Integration Analysis of m6A Related Genes in Skin Cutaneous Melanoma and the Biological Function Research of the SPRR1B
Source: Front Oncol. 2021 Oct 19;11:729045. doi: 10.3389/fonc.2021.729045 (PMC8560968; doi:10.3389/fonc.2021.729045)
Supplement: Supplementary file 2 [file Table_1.docx]

Supplementary Table 1. The first 20 gene results differ between elevated and down-regulated respectively genes in metastatic cancer.

| **Up-regulated Gene** | **log2 Fold Change** | **Value of p** | **Down-regulated genes** | **log2 Fold Change** | **Value of p** |
| --- | --- | --- | --- | --- | --- |
| HTN3 | 4.1776587 | 3.57E-13 | LCE3D | -6.238229184 | 3.61E-22 |
| CSN2 | 2.516527508 | 0.001723505 | KRT6C | -6.010031093 | 2.95E-27 |
| SPATA31C2 | 2.500708926 | 7.17E-10 | SPRR1B | -5.951972362 | 1.57E-27 |
| CDH10 | 2.46575675 | 1.60E-09 | LGALS7 | -5.873400125 | 1.14E-21 |
| SPANXB1 | 2.463358333 | 1.30E-06 | KRT6B | -5.872426033 | 2.10E-41 |
| LINC00347 | 2.198004927 | 0.000164376 | KRT6A | -5.868183674 | 5.39E-37 |
| PRH2 | 2.121660851 | 6.19E-19 | KRT2 | -5.68672604 | 1.43E-42 |
| AC109492.1 | 2.107615353 | 2.30E-05 | KRT16 | -5.664274271 | 1.79E-33 |
| AC020571.1 | 1.955986999 | 3.87E-16 | KRT14 | -5.450822732 | 8.23E-39 |
| PRB3 | 1.950437773 | 8.73E-15 | KRT17 | -5.002462853 | 3.43E-44 |
| LINC01425 | 1.942467222 | 3.01E-05 | DEFB4A | -4.989693342 | 4.86E-13 |
| AC092675.1 | 1.922154399 | 3.11E-06 | SFN | -4.880753996 | 5.23E-44 |
| TMEFF2 | 1.884198824 | 4.15E-08 | WFDC12 | -4.880353997 | 6.02E-15 |
| FGF5 | 1.831061036 | 4.74E-08 | LINC01527 | -4.86254116 | 1.66E-12 |
| BEX1 | 1.826705324 | 6.55E-09 | SPRR4 | -4.79263007 | 4.75E-10 |
| IGF2BP1 | 1.806293553 | 5.95E-09 | CDSN | -4.698772691 | 1.29E-17 |
| AC079062.1 | 1.799890647 | 5.59E-06 | IVL | -4.66936772 | 2.00E-23 |
| PRAC1 | 1.73832941 | 1.40E-05 | KRT5 | -4.611168386 | 1.30E-32 |
| GAD2 | 1.717563853 | 0.000582892 | C10orf99 | -4.550561001 | 4.24E-15 |
| PRSS56 | 1.717399114 | 4.79E-05 | LCE2C | -4.541140553 | 1.66E-06 |
