# Supplementary material for: Integration Analysis of m6A Related Genes in Skin Cutaneous Melanoma and the Biological Function Research of the SPRR1B
Source: Front Oncol. 2021 Oct 19;11:729045. doi: 10.3389/fonc.2021.729045 (PMC8560968; doi:10.3389/fonc.2021.729045)
Supplement: Supplementary file 3 [file Table_2.docx]

| **Location of TMA** | **sex** | **diagnosis** | **IGF2BP3 (cytoplasm)** | | **SPRR1B (cytoplasm)** | | |
| --- | --- | --- | --- | --- | --- | --- | --- |
|  |  |  | **Staining intensity** | **positive rate** | | **Staining intensity** | **positive rate** |
| A01 | Female | Left foot, melanoma | 1~2 | 95 | | 1~3 | 80 |
| A02 | Male | Small bowel, metastatic melanoma | 1 | 90 | | 0 | 0 |
| A03 | Female | Foot, primary melanoma | 0.5~1 | 60 | | 1~2 | 5 |
| A04 | Male | Rectum, metastatic melanoma | 1~2 | 95 | | 0 | 0 |
| A05 | Male | Inner canthus in the right eye， metastatic melanoma | No tumor in the TMA | | | No tumor in the TMA | |
| A06 | Female | Rectum， metastatic melanoma | 1~2 | 95 | | 1 | <5 |
| A07 | Female | Plantar dermis，primary melanoma | 1~2 | 95 | | 0.5~1 | 40 |
| A08 | Female | Eye socket， metastatic melanoma | 2 | 95 | | 0 | 0 |
| A09 | Female | Toe，primary melanoma | 1 | 90 | | 0.5~1 | <5 |
| B01 | Female | Left foot，primary melanoma | 1~2 | 95 | | 0 | 0 |
| B02 | Male | Small bowel, metastatic melanoma | 1 | 85 | | 0 | 0 |
| B03 | Female | Foot,primary melanoma | 0.5~1 | 70 | | 0.5~1 | 30 |
| B04 | Male | Rectum, metastatic melanoma | 1~2 | 95 | | 0.5~1 | 5 |
| B05 | Male | Inner canthus in the right eye， metastatic melanoma | 2 | 95 | | 0 | 0 |
| B06 | Female | Rectum， metastatic melanoma | 1~2 | 95 | | 0 | 0 |
| B07 | Female | Plantar dermis, primary melanoma | 2 | 95 | | 0.5~1 | 70 |
| B08 | Female | Eye socket， metastatic melanoma | 2~3 | 95 | | 2~3 | 90 |
| B09 | Female | Toe, primary melanoma | 1 | 95 | | 0.5~1 | 10 |
| C01 | Female | Normal skin | 0 | 0 | | 0 | 0 |
| C02 | Female | Normal skin | 0 | 0 | | 0 | 0 |
| C03 | Female | Normal skin | 0.5~1 | 50 | | 0.5~1 | 50 |
| C04 | Female | Normal skin | 0 | 0 | | 0 | 0 |
| C05 | Female | Normal skin | 1 | 60 | | 1~2 | 55 |
| C06 | Female | Normal skin | 0 | 0 | | 0 | 0 |
| C07 | Female | Normal skin | 0 | 0 | | 0 | 0 |
| C08 | Female | Normal skin | 1 | 55 | | 0.5~1 | 60 |
| C09 | Female | Normal skin | 0.5~1 | 70 | | 0.5~1 | 5 |
| D01 | Female | Normal skin | 0 | 0 | | 0 | 0 |
| D02 | Female | Normal skin | 0.5~1 | 65 | | 0.5~1 | 40 |
| D03 | Female | Normal skin | 1 | 80 | | 0.5~1 | 85 |
| D04 | Female | Normal skin | 0 | 0 | | 0 | 0 |
| D05 | Female | Normal skin | 0.5~1 | 80 | | 1~3 | 70 |
| D06 | Female | Normal skin | 0 | 0 | | 0 | 0 |
| D07 | Female | Normal skin | 0 | 0 | | 0 | 0 |
| D08 | Female | Normal skin | 0 | 0 | | 0 | 0 |
| D09 | Female | Normal skin | 0.5~1 | 65 | | 0.5~1 | 5 |

**Supplementary Table 2**. The results of TMA
